# Beliefs of parents in Kuwait about thirdhand smoke and its relation to home smoking rules: A cross-sectional study

**DOI:** 10.18332/tid/140090

**Published:** 2021-08-30

**Authors:** Kawthar Shehab, Ali H. Ziyab

**Affiliations:** 1 Department of Community Medicine and Behavioral Sciences, Faculty of Medicine, Kuwait University, Kuwait City, Kuwait

**Keywords:** parents, beliefs, Kuwait, thirdhand smoke, home smoking ban

## Abstract

**INTRODUCTION:**

Thirdhand smoke (THS) is the toxic residue of tobacco smoke that persists long after tobacco smoking on the clothing and hair of smokers and in the surrounding environment. This study aimed to assess parents’ beliefs about THS in terms of harm and persistence in the environment and evaluate associations between parents’ THS beliefs and home smoking rules.

**METHODS:**

A sample of parents living in Kuwait were enrolled in a cross-sectional study. Parents reported home smoking rules (strict, partial, and no, home smoking ban) and completed the 9-item Beliefs About ThirdHand Smoke (BATHS) scale that assessed parents’ overall, health, and persistence beliefs about THS. Associations between the quartiles of THS overall, health, and persistence beliefs scores and home smoking rules were evaluated using a modified Poisson regression, and adjusted prevalence ratios (APR) and 95% confidence intervals (CI) were estimated.

**RESULTS:**

In total, 536 parents (404 females) were enrolled in the study, with 42.0% (n=225) and 43.6% (n=234) reporting a strict or partial home smoking ban, respectively. The prevalence of a strict home smoking ban was higher among never smokers than among ever smokers (49.1% vs 25.2%, p<0.001). The majority of participants indicated that THS exposure harms the health of children (67.2%) and adults (60.6%) and THS residue could remain for days in the environment (58.9%). The prevalence of a strict home smoking ban increased as THS overall (APR_Q4 vs Q1_ = 1.48; 95% CI: 1.12–1.96), health (APR_Q4 vs Q1_ = 1.22; 1.02–1.45), and persistence (APR_Q4 vs Q1_ = 1.55; 1.17–2.05) beliefs scores increased.

**CONCLUSIONS:**

Parents’ harm and persistence beliefs about THS were associated with enforcing a strict home smoking ban, which provides a safer environment for children and non-smokers. Therefore, tobacco prevention programs need to incorporate educational messages about the harm of THS in an attempt to promote smoke-free homes.

## INTRODUCTION

Thirdhand smoke (THS) has been recognized as a public health hazard that is distinct from secondhand smoke (SHS). Matt et al.^1^ defined THS in 2011 as ‘residual tobacco smoke pollutants that remain on surfaces and in dust after tobacco has been smoked, are re-emitted into the gas phase, or react with oxidants and other compounds in the environment to yield secondary pollutants’. On the other hand, secondhand smoke (SHS), an established and serious health hazard^2,3^, consists of a ‘mixture of the sidestream smoke (i.e. smoke emitted from the burning cigarette, pipe, or cigar) and the mainstream smoke exhaled from the lungs of smokers’^1^. Hence, children and non-smoker adults experience SHS exposure by involuntary inhalation of sidestream and exhaled mainstream smoke. Exposure to THS occurs after cigarettes/tobacco products have been extinguished through involuntary inhalation, ingestion, or dermal absorption of residual tobacco smoke pollutants that persist long after the clearing of SHS^4,5^. Collectively, as proposed by Protano and Vitali^4^, the term ‘environmental tobacco smoke’ (ETS) or ‘passive smoking’ should be used to describe both SHS and THS (i.e. exposure to tobacco smoke pollutants during [SHS] and after [THS] tobacco smoking). SHS and THS are distinct and major sources of involuntary exposure to tobacco smoke pollutants that may predispose children and non-smoker adults to adverse health effects.

Previous studies have shown that THS is present on the smokers’ clothes, skin, and hair, as well as on household surfaces such as walls, beds, couches, carpets, and desks^6-8^. Contaminants of THS include carcinogenic and toxic compounds such as polycyclic aromatic hydrocarbons, nicotine, cotinine, phenol, cresols, formaldehyde, and tobacco-specific nitrosamines^1,9^. Hence, THS exposure is not hazard-free. In addition to non-smoking adults, children are most susceptible to THS exposure because they spend more time indoors and have increased hand-to-mouth behavior, increasing their chances of exposure^10,11^. Given their immature respiratory and immune systems, children are more vulnerable and sensitive to the adverse effects of THS exposure in places where smoking is allowed, especially in homes^11,12^. Emerging studies have highlighted the potential harm of THS exposure and its association with adverse health effects. Experimental animal model studies have shown that exposure to THS is associated with reduced body weight in neonatal mice, metabolomic alterations in human reproductive cell lines, and changes in immunological parameters in the blood of experimental mice^13,14^. Also, it has been shown that experimentally exposing human cell lines to THS pollutants is associated with major DNA damage^15^. Martins-Green et al.^16^ reported a possible association between THS exposure and hyperactivity-related behavior in THS-exposed mice. The negative health impact of THS was further demonstrated by Wang et al.^17^ who have shown that THS exposure during pregnancy is associated with increased postpartum depression risk among Chinese women. Nonetheless, more studies are needed to further elucidate the health effects of THS exposure.

Increased knowledge and beliefs about the harm of ETS exposure have been reported to be associated with protective behaviors, such as smoke-free home rules and avoiding exposure to ETS^18-21^. Protano et al.^22^ have assessed the association between home-smoking rules and urinary cotinine concentrations in children, and reported that ETS exposure level (as measured by urinary cotinine concentration) increased among children as home-smoking rules were more permitting. For example, ETS exposure level was lowest among children not living with smoker(s), and was highest among children living with smoker(s) who smoke at home even in the presence of the child^22^. Regarding THS, few prior studies have examined the association between THS knowledge and beliefs with smoking-related preventive measures. A study by Winickoff et al.^23^ showed that beliefs about THS health effects on children were independently linked with strict home smoking bans. Another study concluded that beliefs of THS harm to children were associated with strict implementation of smoke-free home and car measures and increased attempts to quit smoking^24^. The findings of the aforementioned studies indicate that THS harm beliefs may be a critical factor in encouraging home smoking bans. Participants in a qualitative study believed that being educated about THS harms would motivate people to adopt smoke-free home rules^25^. Nevertheless, THS is scarcely discussed in health promotion strategies and policies, and knowledge and beliefs about THS are not widely assessed globally at the population level.

In a nationally representative study sample in Kuwait, the prevalence of current (any use in the past 30-days) cigarette smoking among adults was estimated to be 39.2% among men and 3.3% among women^26^. Moreover, a study conducted among high school students in Kuwait showed that 26.4%, 25.1%, and 20.9%, of the study participants were current e-cigarette users, conventional cigarette smokers, and hookah smokers, respectively^27^. Moreover, in Kuwait, 45.8% of middle school students and 51.6% of high school students were reported to be exposed to household ETS^28^. As a new source of passive smoking, 32.0% of high school students in Kuwait reported being exposed to electronic cigarette aerosols (vapor) in households^27^. Aerosols from electronic cigarettes differ in their constituents compared to combustible tobacco smoke; however, they still contain ultrafine particles such as volatile carbonyls, reactive oxygen species, furans, formaldehyde, and metals (cadmium, lead, nickel, tin, copper, chromium) that have been shown to adversely affect health^29-32^. In terms of the relevance of electronic cigarette aerosols to THS, a prior study has shown that electronic cigarettes emit submicron and ultrafine particles that can persist in the exposed environment and hence expose others to THS pollutants^33^. Collectively, such an elevated passive smoking exposure among children in Kuwait indicates that household THS exposure is substantial as well. To our knowledge, no prior studies have investigated parents’ beliefs in Kuwait about THS harm, which is an important element that may help reduce the negative health impact of tobacco smoke exposure in children and non-smoker adults. Therefore, this study aimed to assess parents’ beliefs about THS in terms of harm and persistence in the environment and to evaluate associations between parents’ THS beliefs and home smoking rules.

## METHODS

### Study setting, design, and participants

A cross-sectional study was conducted by enrolling parents residing in Kuwait (n=536; aged ≥18 years) using a web-based survey that was disseminated using email and social media platforms, including Twitter, Instagram, and WhatsApp. Parents with at least one child aged <18 years were invited to participate in the study. The enrollment of subjects started on 18 December 2020 and ceased on 27 January 2021. The snowball sampling technique, a non-probability sampling method that yields a convenience sample, was used to recruit participants. The study was approved by the Health Sciences Center Ethics Committee of Kuwait University (No. VDR/EC/3687). Completion of the questionnaire by the participants was deemed as informed consent to participate. The study was conducted in accordance with the principles and guidelines of the Declaration of Helsinki for medical research involving human subjects.

### Study questionnaire and variable definitions

The study questionnaire, designed to be self-completed by parents, gathered information on sociodemographic data, lifestyle factors, home smoking rules, and beliefs about THS. Participants self-reported their age in years (18–34, 35–44, or ≥45; this age categorization was applied to closely resemble the distribution of the measured continuous age variable in the total study sample), highest educational attainment (high school degree or less, diploma degree, Bachelor’s degree, or higher education/graduate degree), total monthly household income in Kuwaiti Dinar (KWD: ≤1500, 1501–3000, or ≥3001; this categorization was based on the results of the ‘Income and Expenditure Household Survey’ by the Central Statistical Bureau, Kuwait^34^), housing type (apartment, floor-through apartment, or house), nationality (Kuwaiti or non-Kuwaiti), and current governorate of residence (Al-Asimah, Hawalli, Mubarak Al-Kabeer, Farwaniya, Ahmadi, or Jahra). Moreover, parents were asked to report whether they had ever smoked tobacco products. Those who had ever smoked were further asked whether they had used any tobacco product (i.e. cigarettes, waterpipe, electronic cigarettes, or other) in the past 30 days. Hence, smoking status was ascertained as never, former (ever smoked tobacco product, but not in the past 30 days), and current (smoked tobacco product in the past 30 days). Due to the limited number of participants that were classified as former smokers (n=28), we combined former and current smokers in a single group and reported them as ever smokers.

Home smoking rules were assessed by asking parents to choose which statement best describes the ‘rules’ of smoking inside their home: ‘nobody can smoke (smoking is not allowed) inside the home’, ‘you can only smoke in some places inside the home’, ‘you can smoke anywhere (there are no rules) inside the home’, or ‘don't know/not sure’^23^. Parents who reported that smoking is not allowed in any part of their home were classified as having strict rules prohibiting smoking in their home. Parents who reported that smoking was allowed in some parts inside their home were classified as having partial rules prohibiting smoking in their home. Parents who reported that smoking is allowed anywhere inside their home or were unaware or unsure of smoking rules in their home, were classified as having no rule prohibiting smoking inside their home. Hence, the home smoking rule variable was categorized as: strict home smoking ban, partial home smoking ban, and no home smoking ban.

The Beliefs About ThirdHand Smoke (BATHS) scale, a standardized 9-item scale, was used to assess parents’ overall beliefs about THS (referred to as: ‘THS overall beliefs score’)^35^. Moreover, of the nine items, five items were related to beliefs about THS impact on health (referred to as: ‘THS health beliefs score’) and four items were related to beliefs about THS persistence in the environment (referred to as: ‘THS persistence beliefs score’)^35^. The THS health belief items included the following statements^35^: ‘breathing air in a room today where people smoked yesterday can harm the health of infants and children’, ‘breathing air in a room today where people smoked yesterday can harm the health of adults’, ‘particles in rooms where people smoked yesterday can cause cancer’, ‘after smoking cigarette, smoke particles on skin, hair, and clothing can passed on to others through touch’, and ‘after touching surfaces where cigarette smoke has settled, particles can enter the body through skin’. The THS persistence belief items included the following statements: ‘smoke particles can remain in a room for days’, ‘smoke particles can remain in a room for weeks’, ‘smoke particles get absorbed into furniture and walls’, and ‘opening windows or using air conditioners does not eliminate all smoke particles in a room’. For each of the nine items, response options were measured on a 5-point Likert scale, where parents were asked to indicate whether they strongly disagree, disagree, not sure, agree, or strongly agree. The responses were coded 1 through 5, with 1 corresponding to a strongly disagree response and 5 corresponding to a strongly agree response. The ‘THS overall beliefs score’, based on 9 items, can range from 9 to 45; the ‘THS health beliefs score’, based on 5 items, can range from 5 to 25; and the ‘THS persistence beliefs score’, based on 4 items, can range from 4 to 20. Higher scores indicate stronger beliefs about the harm and persistence of THS.

### Statistical analysis

Analyses were conducted using IBM^®^ SPSS Statistics for Windows, Version 25.0 (IBM Corp., Armonk, NY, USA) and SAS 9.4 (SAS Institute, Cary, NC, USA). The statistical significance level was set to α=0.05, for all association analyses. Descriptive analyses were conducted to calculate the frequencies and proportions of the categorical variables. The THS overall, health, and persistence beliefs scores, non-normally distributed quantitative variables, were described by calculating the median and interquartile range (IQR). Chi-squared (χ^2^) tests were used to assess whether the prevalence of home smoking rules (i.e. strict home smoking ban, partial home smoking ban, and no home smoking ban) differed depending on sociodemographic and lifestyle factors. Non-parametric tests were used to determine whether the median of the THS overall, health, and persistence beliefs scores differed across groups of categorical variables. The Wilcoxon rank sum test was used to compare the medians of two groups, and the Kruskal-Wallis test was used to determine whether the medians of three or more groups differed. Moreover, the THS overall, health, and persistence beliefs scores were categorized using quartiles (Q), where the quartile 1 (Q1) group included individuals with the lowest scores and the quartile 4 (Q4) group included individuals with the highest scores. Associations between quartiles of the THS overall, health, and persistence beliefs scores (independent variables) and home smoking rules (outcome variable: strict home smoking ban vs no home smoking ban; and partial home smoking ban vs no home smoking ban) were assessed by applying a modified Poisson regression with robust variance estimation to estimate adjusted prevalence ratios (APRs) and their 95% confidence intervals (CIs)^36^. Variables that demonstrated a possible association with THS overall, health, and persistence beliefs scores (independent variables) and/or home smoking rules (outcome variable) in bivariate analyses (i.e. p≤0.2, as suggested by Maldonado and Greenland^37^) were simultaneously entered into the multivariable regression models. A separate regression model was used to assess the association between each of the three THS beliefs score (i.e. overall, health, and persistence THS beliefs scores) and home smoking rules, while adjusting for the effect of the aforementioned confounders.

## RESULTS

In total, 536 parents residing in Kuwait with at least one child aged <18 years participated in the current study, of whom 132 (24.6%) were males and 404 (75.4%) were females (Table 1). The median (IQR) age of the study participants was estimated to be 34.0 (29–40) years. The majority of participants were aged 18–34 years (50.9%) and reported to have attained a Bachelor’s degree (57.6%). Ever smoking a tobacco product was reported by 29.7% of the study participants, with the prevalence of current smoking being 24.4% (131/536; i.e. excluding 28 former smokers). The vast majority of the participants were of Kuwaiti nationality (92.0%). Of the total study sample, 42.0% reported having a strict home smoking ban (i.e. smoking inside their home is prohibited), 43.6% reported having a partial home smoking ban (i.e. smoking inside their home is allowed in some places), and 14.4% reported that smoking is allowed inside their home with no restrictions (Table 1). No significant difference according to sex was observed in the prevalence of home smoking bans (p=0.749). Parents aged 18–34 years reported the lowest prevalence of strict home smoking ban (35.9%, p=0.056) compared to parents aged 35–44 years (48.9%) and ≥45 years (47.2%) (Table 1). Whereas, the highest prevalence of partial home smoking ban was seen among young parents aged 18–34 years (49.1%). Moreover, the prevalence of strict home smoking ban was higher among never smokers than among ever smokers (49.1% vs 25.2%, p<0.001), whereas partial home smoking ban was higher among ever smokers than among never smokers (59.1% vs 37.1%). Strict home smoking bans were most prevalent among participants reporting a monthly household income of ≥3001 KWD (52.2%), subjects with a higher education degree (i.e. postgraduate degree, 57.4%), and those living in a house (49.3%) (Table 1).

**Table 1:** Distribution of demographic characteristics of study participants in the total study sample and according to home smoking rules

	*Total sample % (n)*	*Home smoking rules, % (n/total)*			
		*Strict home smoking ban*	*Partial home smoking ban*	*No home smoking ban*	*p* ^†^
**Overall**	100 (536)	42.0 (225/536)	43.6 (234/536)	14.4 (77/536)	-
**Sex**					
Male	24.6 (132)	40.9 (54/132)	46.2 (61/132)	12.9 (17/132)	0.749
Female	75.4 (404)	42.3 (171/404)	42.8 (173/404)	14.9 (60/404)	
**Age** (years)					
18–34	50.9 (273)	35.9 (98/273)	49.1 (134/273)	15.0 (41/273)	0.056
35–44	32.5 (174)	48.9 (85/174)	36.8 (64/174)	14.3 (25/174)	
≥45	16.6 (89)	47.2 (42/89)	40.4 (36/89)	12.4 (11/89)	
**Education level**					
High school degree or less	8.2 (44)	34.1 (15/44)	43.2 (19/44)	22.7 (10/44)	0.045
Diploma degree^#^	21.5 (115)	34.8 (40/115)	50.4 (58/115)	14.8 (17/115)	
Bachelor’s degree	57.6 (309)	42.4 (131/309)	42.4 (131/309)	15.2 (47/309)	
Higher education degree	12.7 (68)	57.4 (39/68)	38.2 (26/68)	4.4 (3/68)	
**Smoking status**					
Never smoked	70.3 (377)	49.1 (185/377)	37.1 (140/377)	13.8 (52/377)	<0.001
Ever smoked (former/current)*	29.7 (159)	25.2 (40/159)	59.1 (94/159)	15.7 (25/159)	
**Monthly household income** (KWD)					
≤1500	33.4 (179)	40.2 (72/179)	46.9 (84/179)	12.9 (23/179)	0.069
1501–3000	45.5 (244)	38.5 (94/244)	43.9 (107/244)	17.6 (43/244)	
≥3001	21.1 (113)	52.2 (59/113)	38.1 (43/113)	9.7 (11/113)	
**Type of housing**					
Apartment	55.0 (295)	36.6 (108/295)	46.8 (138/295)	16.6 (49/295)	0.044
Floor-through apartment	18.9 (101)	47.5 (48/101)	43.6 (44/101)	8.9 (9/101)	
House	26.1 (140)	49.3 (69/140)	37.1 (52/140)	13.6 (19/140)	
**Governorate of residence**					
Al-Asimah	21.3 (114)	50.0 (57/114)	34.2 (39/114)	15.8 (18/114)	0.262
Hawalli	35.8 (192)	44.3 (85/192)	42.7 (82/192)	13.0 (25/192)	
Mubarak Al-Kabeer	22.0 (118)	35.6 (42/118)	50.0 (59/118)	14.4 (17/118)	
Farwaniya	11.2 (60)	41.7 (25/60)	41.7 (25/60)	16.6 (10/60)	
Ahmadi	5.8 (31)	35.5 (11/31)	48.4 (15/31)	16.1 (5/31)	
Jahra	3.9 (21)	23.8 (5/21)	66.7 (14/21)	9.5 (2/21)	
**Nationality**					
Kuwaiti	92.0 (493)	41.6 (205/493)	44.2 (218/493)	14.2 (70/493)	0.673
Non-Kuwaiti	8.0 (43)	46.5 (20/43)	37.2 (16/43)	16.3 (7/43)	

KWD: 100 Kuwaiti Dinar about US$333.

# Refers to 2 years post high school education.

* Only 28 participants were classified as former smokers and were thus combined with current smokers.

† Calculated using chi-squared tests.

**Figure 1:**
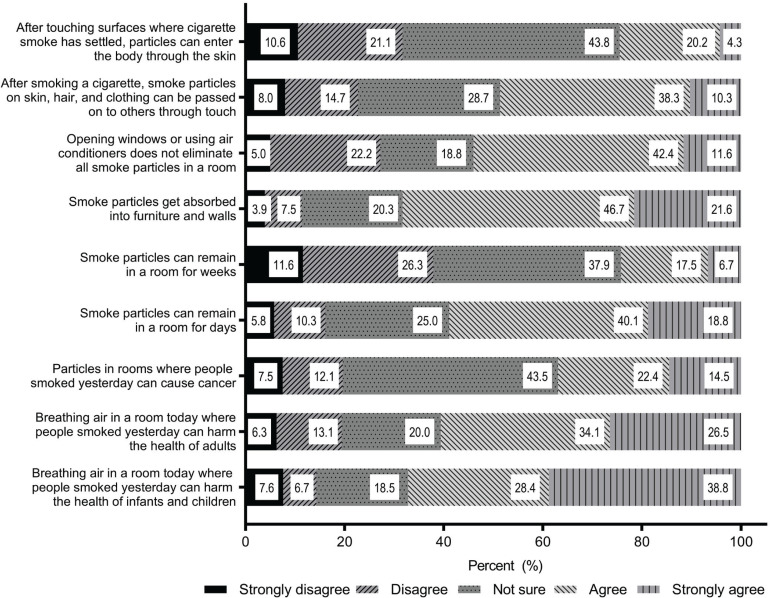
Parents’ response to each of the 9-items assessed in the Beliefs About Thirdhand Smoke (BATHS) scale

Figure 1 shows parents’ responses to each of the 9-items used to assess beliefs about THS. A large proportion of parents agreed or strongly agreed that THS harms both the health of children (67.2%) and adults (60.6%). Regarding whether THS particles in rooms exposed to smoking can cause cancer, 36.9% of participants agreed or strongly agreed with this statement. Moreover, a high proportion of participants agreed or strongly agreed that smoke particles could remain for days in a room (58.9%), whereas only 24.2% of participants believed that smoke particles could remain for weeks. The belief that smoke particles get absorbed into the furniture and walls was reported by 68.3% of the participants. Only 24.5% of participants agreed or strongly agreed that dermal absorption of smoke particles through touching surfaces exposed to cigarette smoke was possible. In contrast, 48.6% of participants believed that smoke particles on the skin, hair and clothing of smokers could be passed to others through touch. More than half (54.0%) of the participants agreed or strongly agreed that opening windows/using air conditioners does not eliminate all indoor smoke particles (Figure 1).

Results of bivariate analysis between THS overall, health, and persistence beliefs scores and participants’ characteristics and lifestyle factors are shown in Table 2. The median THS overall and health beliefs scores were higher among female than male participants, but males and females had comparable THS persistence beliefs scores. Moreover, the THS overall, health, and persistence beliefs scores differed according to the participants’ education level and monthly household income. On average, never smokers had higher THS overall (32.0 vs 29.0, p<0.001), health (18.0 vs 16.0, p<0.001), and persistence (14.0 vs 13.0, p<0.001) beliefs scores than ever smokers. Participants living in an apartment had lower THS persistence beliefs score than those living in a floor-through apartment or house (Table 2).

**Table 2:** Distribution of thirdhand smoke (THS) overall, health, and persistence beliefs scores across characteristics of participants

	*THS overall beliefs score*		*THS health beliefs score*		*THS persistence beliefs score*	
	*Median (IQR)*	p*	*Median (IQR)*	p*	*Median (IQR)*	p*
**Overall**	31.0 (8.0)	-	17.0 (5.0)	-	14.0 (3.0)	-
**Sex**						
Male	30.0 (9.5)	0.066	17.0 (6.5)	0.038	14.0 (4.0)	0.381
Female	31.0 (7.0)		18.0 (5.0)		14.0 (3.0)	
**Age** (years)						
18–34	31.0 (8.0)	0.163	17.0 (6.0)	0.239	13.0 (4.0)	0.024
35–44	31.0 (7.0)		18.0 (6.0)		14.0 (3.0)	
≥45	32.0 (7.0)		18.0 (5.0)		14.0 (4.0)	
**Education level**						
High school degree or less	31.0 (8.5)	0.001	18.0 (5.5)	0.003	14.0 (4.0)	0.003
Diploma degree^#^	30.0 (8.0)		17.0 (6.0)		13.0 (5.0)	
Bachelor’s degree	31.0 (8.0)		17.0 (5.0)		14.0 (3.0)	
Higher education degree	33.0 (8.0)		19.0 (5.0)		14.0 (4.0)	
**Smoking status**						
Never smoked	32.0 (7.0)	<0.001	18.0 (4.0)	<0.001	14.0 (4.0)	<0.001
Ever smoked (former/current)	29.0 (10.0)		16.0 (7.0)		13.0 (4.0)	
**Monthly household income** (KWD)						
≤1500	30.0 (7.0)	0.011	17.0 (4.0)	0.009	13.0 (3.0)	0.051
1501–3000	31.0 (9.5)		17.0 (6.0)		14.0 (4.0)	
≥3001	32.0 (6.0)		18.0 (4.0)		14.0 (2.0)	
**Type of housing**						
Apartment	30.0 (7.0)	0.107	17.0 (5.0)	0.124	13.0 (3.0)	0.034
Floor-through apartment	31.0 (8.0)		18.0 (6.0)		14.0 (4.0)	
House	31.0 (7.0)		18.0 (5.0)		14.0 (3.5)	
**Governorate of residence**						
Al-Asimah	31.0 (7.0)	0.639	18.0 (5.0)	0.778	14.0 (3.0)	0.552
Hawalli	31.0 (8.0)		17.0 (5.0)		14.0 (4.0)	
Mubarak Al-Kabeer	31.0 (7.0)		17.0 (6.0)		14.0 (3.0)	
Farwaniya	32.0 (6.0)		18.0 (5.0)		14.0 (4.0)	
Ahmadi	30.0 (10.0)		17.0 (7.0)		13.0 (3.0)	
Jahra	29.0 (10.0)		16.0 (4.0)		13.0 (4.0)	
**Nationality**						
Kuwaiti	31.0 (6.0)	0.655	17.0 (5.0)	0.680	14.0 (3.0)	0.925
Non-Kuwaiti	30.0 (9.0)		17.0 (6.0)		14.0 (3.0)	

KWD: 100 Kuwaiti Dinar about US$333. IQR: interquartile range; THS: thirdhand smoke.

# Refers to 2 years post high school education.

* Calculated using the Wilcoxon rank sum test when comparing the medians of two groups, and the Kruskal-Wallis test when comparing medians of three or more groups.

Table 3 shows the associations between quartiles of THS overall, health, and persistence beliefs scores and home smoking rule. In general, prevalence of strict home smoking bans increased as the quartile of THS overall, health, and persistence beliefs scores increased. For example, compared to parents in quartile 1 of the respective THS beliefs score, parents in quartile 4 of the THS overall (60.6% vs 25.2%), health (59.3% vs 32.0%), and persistence (53.8% vs 25.0%) beliefs scores had higher prevalence of strict home smoking bans. Adjusted analysis showed that higher THS overall beliefs score to be associated with higher prevalence of having a strict home smoking ban (APR_Q4 vs Q1_ = 1.48; 95% CI: 1.12–1.96) (Table 3). Similarly, higher THS health (APR_Q4 vs Q1_ = 1.22; 95% CI: 1.02–1.45) and persistence (APR_Q4 vs Q1_ = 1.55; 95% CI: 1.17–2.05) beliefs scores were associated with increased prevalence of a strict home smoking ban (Table 3). THS overall and health beliefs scores were not associated with partial home smoking ban prevalence; however, higher THS persistence beliefs score was associated with increased prevalence of partial home smoking ban (APR_Q4 vs Q1_ = 1.23; 95% CI: 1.04–1.45) (Table 3).

**Table 3:** Associations between thirdhand smoke (THS) overall, health, and persistence beliefs scores quartiles and home smoking rules

*Independent variables^†^*	*Total sample*		*Strict home smoking ban vs No home smoking ban*			*Partial home smoking ban vs No home smoking ban*		
	*n*	*Median (IQR)*	*% (n)*	*Unadjusted PR (95% CI)*	Adjusted PR* (95% CI)	*% (n)*	*Unadjusted PR (95% CI)*	Adjusted PR* (95% CI)
**THS overall beliefs score**								
Quartile 1	123	22.0 (8.0)	25.2 (31)	1 (Ref.)	1 (Ref.)	55.3 (68)	1 (Ref.)	1 (Ref.)
Quartile 2	132	29.0 (2.0)	40.2 (53)	1.29 (0.98–1.69)	1.25 (0.93–1.66)	44.7 (59)	1.01 (0.85–1.21)	1.09 (0.90–1.32)
Quartile 3	144 32.0 (1.0)		40.3 (58)	1.28 (0.98–1.68)	1.26 (0.95–1.68)	44.4 (64)	1.01 (0.85–1.18)	1.07 (0.89–1.28)
Quartile 4	137	37.0 (4.0)	60.6 (83)	1.57 (1.23–2.00)^‡^	1.48 (1.12–1.96)^§^	31.4 (43)	1.08 (0.90–1.29)	1.23 (0.98–1.55)
**THS health beliefs score**								
Quartile 1	125	12.0 (5.0)	32.0 (40)	1 (Ref.)	1 (Ref.)	51.2 (64)	1 (Ref.)	1 (Ref.)
Quartile 2	151	16.0 (2.0)	29.8 (45)	0.95 (0.74–1.23)	0.90 (0.69–1.17)	52.3 (79)	0.99 (0.84–1.17)	1.03 (0.87–1.23)
Quartile 3	110	18.0 (1.0)	46.4 (51)	1.16 (0.93–1.46)	1.10 (0.87–1.38)	39.1 (43)	0.97 (0.79–1.18)	1.01 (0.82–1.24)
Quartile 4	150	21.0 (2.0)	59.3 (89)	1.33 (1.09–1.62)^§^	1.22 (1.02–1.45)^#^	32.0 (48)	1.05 (0.87–1.25)	1.09 (0.91–1.32)
**THS persistence beliefs score**								
Quartile 1	112	9.0 (3.5)	25.0 (28)	1 (Ref.)	1 (Ref.)	53.6 (60)	1 (Ref.)	1 (Ref.)
Quartile 2	137	13.0 (1.0)	40.9 (56)	1.33 (1.00–1.78)^#^	1.26 (0.94–1.70)	43.1 (59)	1.02 (0.84–1.23)	1.08 (0.88–1.32)
Quartile 3	157	14.0 (1.0)	45.2 (71)	1.42 (1.08–1.87)^§^	1.34 (1.00–1.78)^#^	40.8 (64)	1.04 (0.87–1.25)	1.11 (0.92–1.35)
Quartile 4	130	17.0 (3.0)	53.8 (70)	1.65 (1.26–2.14)^‡^	1.55 (1.17–2.05)^§^	39.2 (51)	1.19 (1.00–1.41)^#^	1.23 (1.04–1.45)^#^

PR: prevalence ratio. CI: confidence interval. IQR: interquartile range. THS: thirdhand smoke.

* Adjusted for sex, age, smoking status, education level, housing type, and monthly household income.

† A separate regression model was used to assess the association between the respective THS beliefs score and home smoking rules, while adjusting for the effect of the aforementioned confounders.

# p<0.05.

§ p<0.01.

‡ p<0.001.

## DISCUSSION

This study assessed the beliefs of parents living in Kuwait about THS and determined how these beliefs influence home smoking rules. In total, a strict home smoking ban was reported by 42.0% of the participants, with the prevalence of strict home smoking bans being the lowest among young participants, subjects who reported ever smoking, and those living in an apartment. Whereas, 43.6% of the study participants reported having a partial home smoking ban, with the prevalence of partial home smoking bans being highest among young participants, ever smokers, and those living in an apartment. Only 14.4% of the study participants reported no home smoking bans (i.e. smoking is allowed anywhere in their home). The majority of participants believed that exposure to THS adversely affects the health of children and adults, and that THS particles can persist in the environment for several days. The assessed THS overall, health, and persistence beliefs scores differed according to participants’ education level, household monthly income, and smoking status. In this study, we found that higher THS overall, health, and persistence beliefs scores were associated with an increased prevalence of strict home smoking bans. Only higher THS persistence beliefs score was associated with increased prevalence of partial home smoking bans. These findings provide evidence that educating parents about the harm and persistence of THS may protect children, and even non-smoker adults, from being exposed to THS inside their homes.

The prevalence of strict home smoking bans in the current report (42.0%) was lower than prior studies’ prevalence, with the enforcement of voluntary strict home smoking bans reported to be 61% in homes in Italy^38^, 50% in homes in the United States^35^, and 66.1% in homes in Poland^39^. Similarly, a study based on data from four countries, namely, Canada (67.8%), the United States (60.2%), England (59.2%), and Australia (66.2%), reported higher prevalence of strict home smoking bans than our study^40^. In addition, a study in Spain found that approximately 57.4% of households had complete indoor smoke-free rules^41^. A study based on South African adults reported the prevalence of smoke-free homes to be 62.5%^42^. Moreover, a study from Japan reported that 47.0% of respondents applied comprehensive home and car smoke-free rules^43^. However, a Chinese study reported a lower prevalence of strict home smoking bans (35.2%) than our prevalence^44^.

Findings of the current analysis showed that ever smokers (i.e. former and current smokers) were less likely to implement a smoke-free home rule than never smokers (25.2% vs 49.1%), whereas we found that ever smokers were more likely than never smokers to have partial home smoking bans (59.1% vs 37.1%). In agreement with our findings, the observation of a higher prevalence of smoke-free homes among non-smokers than among smokers has been widely reported. For instance, compared to smokers, non-smokers reported a higher prevalence of smoker-free homes in the United States (88.4% vs 26.7%)^23^, Poland (78.9% vs 18.6%)^39^, Italy (69% vs 32%)^38^, South Africa (71.3% vs 25.9%)^42^, Spain (72.0% vs 28.4%)^41^, and China (81.7% vs 19.3%)^44^. An important observation is that 49.1% of never smokers in our study sample reported a strict home smoking ban, which is much less than the prevalence of smoke-free homes among non-smokers in other settings. For example, based on the aforementioned studies, 88.4% of non-smokers in the United States reported smoke-free home rules^23^. Similarly, 78.9% of non-smokers living in Poland reported smoker-free home rules^39^. Hence, increasing the awareness of non-smokers about the adverse effects of SHS and THS exposure may help increase the prevalence of smoke-free homes in Kuwait. Moreover, we observed that younger parents aged 18–34 years were less likely to implement a strict home smoking rule than older parents. This observation of an association between age and implantation of a smoke-free home rule was not observed in prior studies^23,39^. Furthermore, our results illustrated that subjects who received higher education (i.e. postgraduate degree) were more likely to adopt a strict smoking rule at home than subjects with less educational attainment. This observation is in agreement with a prior study that found college graduates (86.4%) to be more likely to have a strict home smoking ban than subjects with <12 years of formal education (58.7%)^23^. We also observed that subjects living in apartments (36.6%) are less likely to have strict home smoking ban compared to those living in floor-through apartments (47.5%) or houses (49.3%). Such a difference might be explained by the limited/no access to private outdoor space among subjects living in apartments.

Parents’ beliefs about THS were measured using the 9-item BATHS scale, which measures parents’ THS overall, health, and persistence beliefs scores^35^. Most parents agreed or strongly agreed that THS exposure harms the health of children (67.2%) and adults (60.6%). Among a sample of adults living in the United States, 61.0% agreed that THS harms children^23^. However, 91.0% of parents who were living in the United States agreed that THS exposure can harm the health of children^24^. In the current analysis, the calculated THS overall, health, and persistence beliefs scores differed according to the educational attainment of parents, with all of the assessed THS beliefs scores increasing as educational attainment increased. We also observed a positive association between monthly household income and the THS beliefs scores. Similar patterns of associations between parents’ education level and income and THS beliefs scores measured using the BATHS scale were reported among a sample of parents in Shanghai^45^. The aforementioned associations indicate that parents’ socioeconomic status is associated with THS beliefs scores. Moreover, never smokers in our study had higher THS beliefs scores than ever smokers. This relationship between smoking status and THS beliefs has been reported in previous studies^35,45^, which indicates that non-smokers hold stronger harm and persistence beliefs about THS than smokers.

In multivariable analysis, we found independent associations between quartiles of THS overall, health, and persistence beliefs scores with home smoking rules after controlling for the effect of potential confounders. The prevalence of a strict home smoking ban increased as the THS overall, health, and persistence beliefs scores increased. This observation indicates that as parents hold stronger beliefs that THS harms health and persists in the environment, they are more likely to have strict rules banning smoking inside their homes. Moreover, we only found that THS persistence beliefs score to be associated with increased prevalence of partial home smoking ban, whereas THS overall and health beliefs scores were not associated with partial home smoking bans. Overall, such beliefs can protect children and non-smoker adults from the adverse effects of passive smoking, which includes THS exposure. Drehmer et al.^24^ showed that parents who believed that THS exposure is harmful to children were twice as likely to have a strict smoke-free home rule as those who did not believe that THS is harmful to children. Similarly, Winickoff et al.^23^ demonstrated an association between having a belief that THS harms children and the presence of a strict home smoking ban. Haardörfer et al.^35^, using the BATHS scale, showed positive associations between the THS beliefs scores and levels of home smoking bans (i.e. no ban, partial ban, full ban). The findings of associations between beliefs about THS exposure and smoke-free homes should be considered as evidence-based knowledge. Thus, THS needs to be incorporated in health promotion and education campaigns aimed at reducing home smoking. Moreover, more strict health policies are needed to prohibit indoor smoking in public and private settings to protect children and non-smokers from the negative effects of SHS and THS. Policies may designate residential rental properties as ‘smoke-free property’ versus ‘smoke-friendly property’, where non-smoker tenants can have a choice of living in a smoke-free environment.

### Strengths and limitations

Our study provides information about the beliefs of parents in Kuwait about THS for the first time and explored how such beliefs are associated with home smoking rules. Nonetheless, our study has some limitations. The applied snowball sampling technique is a non-random sampling method, which may have yielded a study sample that is not representative of the total parents living in Kuwait with at least one child aged <18 years. Hence, our findings might not be applicable to the entire target population. Moreover, selection bias cannot be eliminated because participants needed access to a smartphone, tablet, or computer to be able to participate and complete the study questionnaire. Nevertheless, in terms of education level and income, our study sample did not substantially deviate from other randomly selected samples in Kuwait. For instance a prior study that enrolled a random sample of parents (n=3864) of high school students in Kuwait reported that 42.7% of mothers and 35.8% of fathers have Bachelor’s degree^46^, which is lower than the reported estimate in the current report (57.6% reported having a Bachelor’s degree). Moreover, the majority of study participants of the aforementioned study sample reported a household income between 1501 and 3000 Kuwaiti Dinars (44.5%)^47^, which is close to what was reported by participants in the current report (45.5% reported a similar income). Hence, the enrolled study sample, to some extent, does represent the target population. The use of the standardized BATHS scale that measured overall, health, and persistence beliefs about THS is a major advantage of our analysis. We have assessed the type of housing (i.e. apartment, floor-through apartment, and house) without inquiring about the size of the living space and the availability of outdoor space (can be used, if available, by smokers), which are important factors that may influence home-smoking rules. The analyzed ‘type of housing’ variable can be a surrogate for the size of indoor space as well as having access to outdoor space; however, such a limited measurement is a further limitation in our study. Moreover, due to only 28 participants being former smokers, we combined them with current smokers in the analysis. Such a combining method may mix the independent effects of former and current smokers. We would think that former smokers might be more likely than current smokers to implement a strict home-smoking ban; however, they are less likely to be as strict as never smokers. Hence, our combined variable (i.e. ever smoked) may overestimate the prevalence of strict home-smoking. Lack of information on car smoking rules is a further limitation of our study.

## CONCLUSIONS

Given that the health effects of THS exposure can be substantial, our study provides novel information about the THS beliefs of parents living in Kuwait and how such beliefs are associated with strict and partial home smoking bans. In this study, we showed that parents who hold strong beliefs about the harm and environmental persistence of THS were more likely to enforce a strict home smoking ban. Such beliefs provide a safer environment for children and non-smoker adults. Hence, health promotion campaigns aimed at reducing the negative effects of passive exposure to tobacco smoke should consider incorporating educational messages about the harm of THS and its persistence in the surrounding environment as an attempt to encourage smoke-free homes.

## Data Availability

The data supporting this research are available from the authors on reasonable request.
